# Self-fertilizing crops: Plant Biotech’s Hardest, Most Rewarding Task

**DOI:** 10.1038/s44319-025-00618-y

**Published:** 2025-11-11

**Authors:** Rene Geurts, Simona Radutoiu

**Affiliations:** 1 Laboratory of Molecular Biology, Department of Plant Science, Wageningen University, Droevendaalsesteeg 1, 6708PB Wageningen, The Netherlands; 2 Department of Molecular Biology and Genetics, Aarhus University, Gustav Wieds vej 10, 8000C Aarhus, Denmark

**Keywords:** Biotechnology & Synthetic Biology, Evolution & Ecology, Plant Biology

## Abstract

For decades, plant biologists have tried to engineer nitrogen fixation into crop plants. With the knowledge from basic research gathered over time, the challenge is now still substantial but not insurmountable.

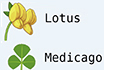

Plants require fixed nitrogen, which is often the growth-limiting nutrient. Legume plants such as beans, lentils and soybeans can overcome a shortage of nitrogen by forming a root nodule endosymbiosis with nitrogen-fixing rhizobium bacteria. These bacteria possess the biochemical machinery to convert atmospheric dinitrogen gas (N_2_) into ammonia (NH_4_^+^), which they exchange with the plant in return for carbon sources and other essential nutrients. Legumes can fix up to 400 kg of nitrogen per hectare, making the rhizobium nodule endosymbiosis an appealing alternative to environmentally harmful chemical nitrogen fertilizers (Box 1).

“Legumes can fix up to 400 kg of nitrogen per hectare, making the rhizobium nodule endosymbiosis an appealing alternative to environmentally harmful chemical nitrogen fertilizers.”

The efficient acquisition of fixed nitrogen by legumes is based on the evolved capacity of root nodules to provide an optimized environment for nitrogen-fixing rhizobium bacteria. Rhizobia are soil-born and compete with other members of the bacterial soil community for survival in the environment around plant roots. Inside root nodules, the bacteria are provided with a nutrient-rich and much less competitive environment, enabling massive bacterial amplification. The bacteria are actually hosted inside the nodule cells, surrounded by a plant-derived membrane— known as the peribacteroid membrane—and behave as transient organelle-like structures. These differentiated bacteria are able to fix nitrogen.

To commit this biochemical function, the bacteria possess a unique enzyme complex, named Nitrogenase. Its activity is enhanced inside the nodules, where the level of oxygen, which is a destabilizing factor for the enzyme complex, is kept low by oxygen-scavenging hemoglobin proteins and structural elements that make the nodule less permeable to oxygen. A complex arsenal of transporters of nutrients, such as dicarboxylates, amino acids and metals, is assembled by the host at the peribacteroid membrane to support bacterial growth and Nitrogenase activity. In return, the bacteria secrete ammonia, which is taken up by the plant. Within nodules, the plant and bacterial metabolisms are thus extensively reprogrammed to facilitate bacterial proliferation, nitrogen fixation, ammonia transport and its assimilation into amino acids.

Ever since the discovery in the late 19th century that legume nodules host rhizobium bacteria responsible for nitrogen fixation, researchers have been intrigued by the idea of transferring this trait to other crops [Burrill and Hansen (1917)]. Each new wave of scientific breakthroughs in the mechanistic understanding of the legume–rhizobium symbiosis has therefore reinvigorated efforts to address this challenge. These breakthroughs can be grouped into at least six major milestones. In the 1970s, researchers uncovered the genetics and biochemistry of Nitrogenase. The 1980s brought the discovery of the first plant genes expressed explicitly in nodules. In the early 1990s, the identification of the rhizobium-secreted lipo-chitooligosaccharide (LCO) signal, known as the nodulation (Nod) factor, revealed how the bacterium activates nodule organogenesis in legumes. From 2000 onward, the genetic dissection of the nodulation trait in model legumes gained momentum. Since 2018, researchers have begun to elucidate the molecular evolutionary trajectory of nodulation. To date, structural biology allows for in-depth analysis of the biochemical properties of key symbiotic proteins.

“Ever since the discovery in the late 19th century that legume nodules host rhizobium bacteria responsible for nitrogen fixation, researchers have been intrigued by the idea of transferring this trait to other crops.”

More than 50 years of biochemical and molecular genetic research have produced an impressive body of knowledge on the molecular mechanisms underpinning nitrogen-fixing nodule symbiosis in legumes. These are unprecedented advances taken for engineering of this trait into non-leguminous crop plants, and such efforts are on a strong trajectory. However, as a *Science* opinion paper already observed in 2016, “Few projects in plant biotechnology are harder, or promise a greater pay-off, than enabling crops to make their own nitrogen fertilizer” [Stokstad (2016)].

Box 1 Chemical nitrogen fertilizer Synthetic nitrogen fertilizer, produced via the Haber–Bosch process, has been one of the most important innovations for global food production. Today, it is estimated to support just under half of the world’s food supply. Since the early 1960s, when average application rates were below 20 kg of nitrogen per hectare of cropland, global fertilizer use has surged dramatically. This increase reflects both the expansion of cropland receiving synthetic fertilizers and rising per-hectare application rates, especially in Asia and Latin America.

Despite this global increase, nitrogen application rates vary widely between regions and countries. On average, farmers worldwide apply ~115 million tons of chemical nitrogen fertilizer to croplands each year. However, only about 35% of this nitrogen is taken up by harvested crops. The remaining 65%—about 75 million tons—of fixed nitrogen is lost, either running off into rivers and lakes, where it contributes to eutrophication, or volatilizing into the atmosphere as nitrous oxide (N₂O). Nitrous oxide is a potent greenhouse gas with a global warming potential 265 times greater than carbon dioxide (CO₂) over a 100-year period.

Nearly all nitrous oxide emissions originate from high-input farming. It is produced by a diverse group of denitrifying soil microbes that are fueled by excess nitrogen fertilizer. Although the ratio of nitrogen in harvested crops to nitrogen applied, known as nitrogen use efficiency (NUE), has improved in recent decades, farmers in many regions still excessively apply nitrogen fertilizer for only marginal yield gains. The environmental consequences of excessive nitrogen use are significant: rising nitrate concentrations in freshwater systems, increased algal blooms, dead zones in coastal waters and elevated greenhouse gas emissions [Ritchie et al, (2022)].

## Factors that hamper progress

Why is engineering the nodulation trait into crop plants so challenging? First and foremost, the nitrogen-fixing nodule symbiosis is a highly complex trait where the microbial infection needs to be rewired against the “natural” state of plants to defend against invading bacteria. Legumes are able to specifically halt their defenses and instead activate root nodule organogenesis after recognition of rhizobium-secreted Nod factor signals. This is accompanied by bacterial intracellular infection, a phenomenon which is rare in plants.

Besides the complexity of the trait, we identify three— partly overlapping—factors that have hindered progress, each of which is surmountable however: focus on model legumes; incomplete understanding of the evolutionary trajectory of the nodulation trait; and the fact that crops that are targets for engineering the nitrogen-fixing trait pose technological challenges to a *“fail fast and repeat*” strategy, which is foundational for successful engineering.

**Figure 1:**
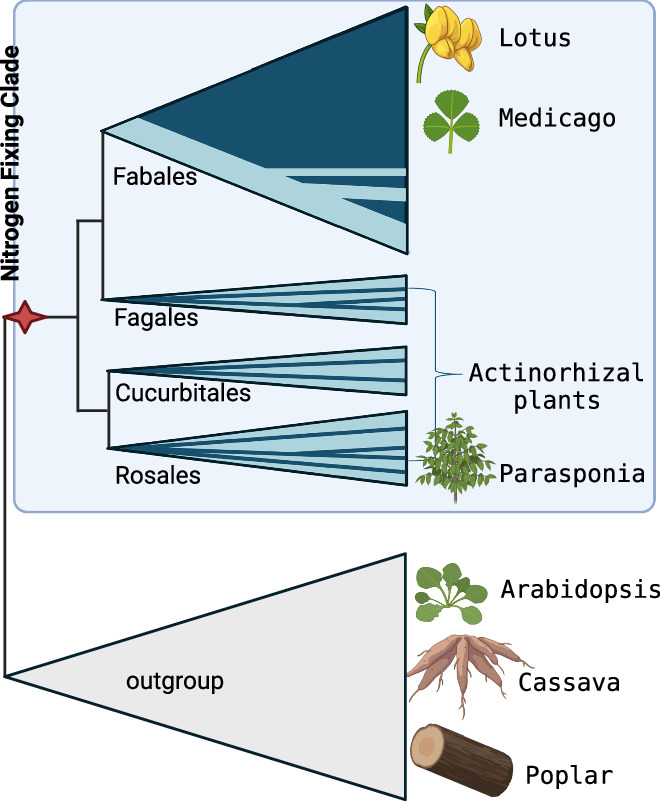
The nitrogen-fixing clade. Four taxonomic orders—Fabales, Fagales, Cucurbitales, and Rosales—together encompass the ten lineages of nodulating plants (marked in dark blue). These nodulating plants share an evolutionary event at the root of the nitrogen-fixing clade (marked with a red asterisk) that was critical for the evolution of nodulation. With the exception of the order Fabales (encompassing legumes), nodulation is rare. Legumes (Fabales) and Parasponia (Rosales) nodulate with rhizobium, whereas Actinorhizal plants form nodulates with *Frankia* bacteria. Arabidopsis, cassava, and poplar represent species of the closest outgroup of the nitrogen-fixing clade (Figure created in BioRender).

Before illustrating how these factors limit engineering efforts, it is helpful to consider the phylogenetic distribution of the nodulation trait (Fig. 1). Nitrogen-fixing root nodules are not exclusive to legumes, nor do all legumes fix nitrogen. The legume family (Fabaceae) is the third-largest family in the plant kingdom, encompassing more than 19,000 species distributed across six subfamilies and close to 800 genera. Despite the predominance of nodulation in the Fabaceae, tens of distinct lineages in this family lack the trait. In addition to the Fabaceae, nine additional families also contain nodulating plants, but collectively these are no more than a few hundred species. With one exception, these non-legume plants do not host rhizobia inside their nodules, but filamentous *Frankia* bacteria from the Actinobacteria phylum; consequently, these nodulation plants are referred to as “actinorhizal”. Interestingly, a small group of five tropical tree species, named *Parasponia*, in the hemp family (Cannabaceae), form nodules with the same LCO-producing rhizobia as legumes do. It shows a mechanistic overlap in the nodulation traits of legumes and *Parasponia* plants.

## Focus on model species

**Figure 2:**
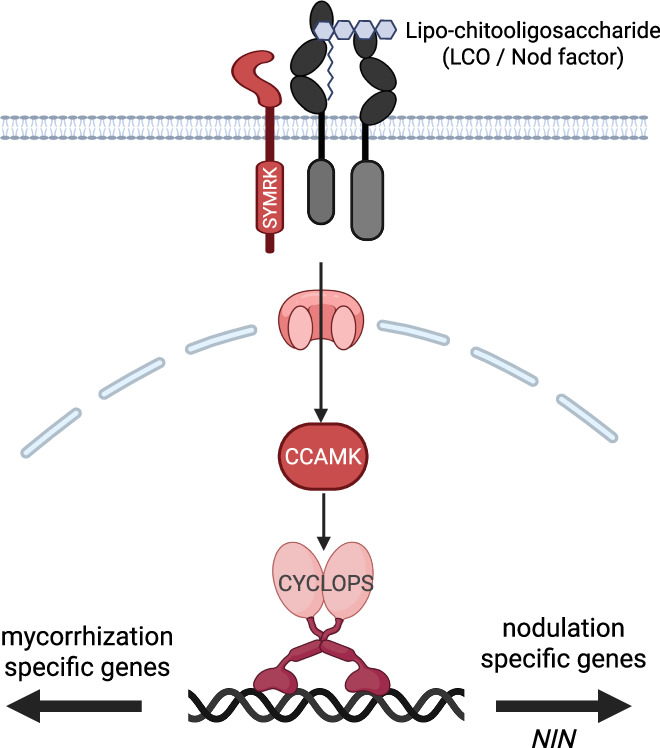
The common symbiosis signaling pathway. Genetic and phylogenomic studies have shown that root nodule symbiosis evolved through rewiring of an ancient signaling pathway that enables arbuscular mycorrhizal (AM) symbiosis. The common symbiosis signaling pathway (marked in red) consists of several signaling components, stretching from the transmembrane receptor SYMRK, ion channels in the nuclear envelope controlling nuclear calcium signaling, a nuclear-localized calcium calmodulin kinase (CCAMK), and the transcriptional regulator CYCLOPS. Note that the nodulation-specific LCO receptors (marked in dark gray) form a complex with SYMRK. Since the vast majority of land plants can form AM symbioses, the presence of this common symbiosis signaling pathway offers a reliable engineering template across many target crops. Note the difference in outcomes between AM- and rhizobium-induced activation of the common symbiosis signaling pathway: rhizobium activates the expression of *NIN*, whereas AM symbiosis does not (Figure created in BioRender).

Before the introduction of model legumes, nodulation genetics was mainly studied in crops such as lucerne, soybean and garden pea. These studies led to key discoveries, including that certain mutants unable to respond to rhizobium also cannot form symbioses with arbuscular mycorrhizal (AM) fungi. AM symbiosis is ancient and widespread among land plants. In the 1980s and 90s, identifying the genes behind non-nodulating mutants was not feasible, though. Today, we know that AM fungi also secrete LCO-type molecules that activate the same symbiosis signaling pathway as rhizobium does, although with a different outcome. In line with this observation, this pathway is referred to as the common symbiosis signaling pathway (Fig. 2).

The cloning of the genes encoding the common symbiosis signaling pathway was made possible by the introduction of model legumes. Inspired by the success of the model plant *Arabidopsis thaliana* (Arabidopsis), the legume research community adopted two species as experimental systems to genetically dissect nodulation: *Lotus japonicus* (Lotus) and *Medicago truncatula* (Medicago). These two models have led to many breakthroughs, resulting in the discovery of nearly 200 nodulation genes, while counting [Roy et al, (2020)].

A relevant highlight of these genetic studies is the discovery that nodule organogenesis evolved by rewiring the lateral root developmental program to couple it with rhizobium Nod factor-triggered activation of the common symbiosis signaling pathway [Schiessl et al, (2019)]. Since lateral root formation is normally induced during nitrogen starvation in both nodulating and non-nodulating plants, the finding that nodule organogenesis uses this program as a chassis represents a major discovery with important implications for engineering strategies in non-legume crops.

While legume model systems have enabled many breakthrough discoveries, they also have limitations. Lotus and Medicago represent only a narrow evolutionary divergence, having split ~40 million years ago, which has resulted in some functional differences in nodulation genes and phenotypic variation in corresponding knockout mutants. One example of such divergence is the LysM-type transmembrane receptor family. These receptors recognize polysaccharides, including rhizobium Nod factors and AM-fungal LCOs, as well as chitin oligomers that act as pathogen-associated molecular patterns (PAMPs) that can trigger an immune reaction. Medicago and Lotus detect distinct structural features of the Nod factors produced by their respective rhizobium microsymbionts, and comparative analysis of their receptors has enabled the identification of critical amino acids that determine specificity [Krönauer and Radutoiu (2021)].

Despite these valuable insights, the comparison of Lotus and Medicago does not fully capture the diversity of the nodulation trait. Broadening the evolutionary spectrum is therefore crucial for designing an engineering blueprint for nodulation. This can be achieved by extending (reverse) genetic studies to legumes from other subfamilies and even to nodulating non-legumes. However, such studies are often technically more challenging, time-consuming and less rewarded by funding agencies and the research community, and thus rarely pursued systematically.

“Broadening the evolutionary spectrum is therefore crucial for designing an engineering blueprint for nodulation.”

A few studies have looked at nodulating non-legumes, from which critical insights have come, too. For instance, these studies have shown that rhizobial Nod factors are perceived by orthologous, but less-diverged LysM-type receptors in *Parasponia* trees. Additionally, the common symbiosis signaling pathway and its direct downstream target, the transcription factor *NODULE INCEPTION* (*NIN*), are essential across all nodulating legume and non-legume species studied. This is a key finding. *NIN* is a nodulation response factor that is transcriptionally activated by the common symbiosis signaling pathway that rewires the lateral root developmental program (Fig. 2). It shows that NIN acts as *the* master regulator in nodulation across all nodulating plant species [Geurts and Huisman (2023)].

The few studies in nodulating non-model species showed that comparative approaches are valuable in guiding the selection of target genes for engineering efforts. Therefore, a comprehensive reverse genetic study of all 200+ nodulation genes across species representing key taxonomic nodes would significantly support the rational design to engineer the nitrogen-fixing nodulation trait.

## Evolutionary uncertainty

In theory, if we would fully understood the key evolutionary innovations that enabled rhizobium or *Frankia* bacteria to induce root nodules in their host plants, we could replicate these mechanisms in non-nodulating species. For a long time, however, the evolutionary trajectory of nodulation remained largely unknown, and is still a matter of debate [Doyle et al, (2025)].

“In theory, if we would fully understood the key evolutionary innovations that enabled rhizobium or *Frankia* bacteria to induce root nodules […], we could replicate these mechanisms in non-nodulating species.”

The ten plant families that contain nodulating species belong to four related taxonomic orders: Fabales, Fagales, Cucurbitales and Rosales. Collectively, these orders form the so-called Nitrogen-Fixing Clade, although it also includes many non-nodulating lineages (Fig. 1). The nodules formed on these species differ in terms of morphology and infection modus of the microsymbiont.

A widely accepted hypothesis proposes that species within the nitrogen-fixing clade are predisposed to gain the ability to nodulate. The evolution of nodules was thought to have occurred independently multiple times, either with rhizobia or *Frankia*. Additionally, it was hypothesized that several lineages, such as those within the legume family, subsequently lost this trait in parallel. However, large-scale genome sequencing of both nodulating and non-nodulating species, followed by detailed evolutionary analyses of nodulation genes, has challenged this view. One of the key arguments against multiple independent gains of nodulation is the widespread presence of pseudogenized versions of essential nodulation genes, most profoundly *NIN*, in virtually all non-nodulating species within the Nitrogen-Fixing Clade. This finding strongly supports an alternative hypothesis: nodules evolved only once at the base of the Nitrogen-Fixing Clade, followed by widespread parallel losses of the trait across various lineages [van Velzen et al, (2019)]. This new perspective raises many unresolved questions and challenges. Among them is the implication that the nodulation trait is more than 100 million years old and may have initially involved *Frankia* rather than rhizobia. What did the earliest nodule-like structures look like? And why did so many lineages lose this seemingly advantageous trait?

Although still disputed and with several open questions, the single-gain, massive-loss hypothesis provides a more transparent framework for identifying key engineering targets through phylogenetic comparative analysis. In fact, only a handful of nodulation genes show consistent patterns of pseudogenization in non-nodulating species within the Nitrogen-Fixing Clade. These genes now serve as primary candidates for engineering infectable protonodules in non-legume crops, where variation in nodule phenotypes informs distinct design strategies.

## Engineering target crops imposes technological challenges

There is no unambiguous model plant that can be used as a chassis for engineering the nodulation trait. The primary model in basic plant research is Arabidopsis, a dicot that can complete its lifecycle in ~6–8 weeks. This short lifecycle is advantageous for trait engineering, which requires iterative rounds of construct design, generation of genetically modified plants and phenotypic testing (Box 2).  Arabidopsis also represents a species of a close outgroup to the Nitrogen-Fixing Clade, which potentially might be instrumental for engineering nodulation. Notably, Arabidopsis recognizes rhizobium Nod factors, which attenuate immune responses typically triggered by pathogenic signals [Liang et al, (2013)]. However, a significant limitation is the loss of the AM symbiosis, a trait absent in most Brassicaceae species. Consequently, the common symbiosis signaling pathway has been genetically eroded [Radhakrishnan et al, (2020)]. It severely complicates the efforts to engineer nodulation, limiting the utility of Arabidopsis for these studies.

Other representatives of the closest outgroup of the Nitrogen-Fixing Clade that are used in engineering efforts are poplar and cassava, both of which could directly benefit from nitrogen-fixing root nodules. Poplar is widely used in agroforestry systems and valued for its versatility and rapid biomass production. Cassava, a tropical crop, thrives in poor soils and withstands drought. It is primarily cultivated by smallholder farmers in Africa and South America for food security; in Asia, it also serves industrial applications. Both species can engage with AM fungi and have established transformation protocols. However, both crops are propagated vegetatively, which might complicate gene stacking and further breeding.

Ultimately, cereals are considered the most relevant target crops [Charpentier and Oldroyd (2010)]. Engineering cereals to fix their own nitrogen could be particularly rewarding, as rice, maize, and wheat underpin global food security and account for a significant share of agricultural nitrogen demand. Adopting any of these species for engineering requires optimization and streamlining of transformation pipelines, possibly supplemented by transient transformation systems, such as protoplast assays, to enable rapid design–build–test–learn cycles (Box 2). Investments in such engineering pipelines will be essential to overcome existing bottlenecks in ongoing engineering approaches.

“Engineering cereals to fix their own nitrogen could be particularly rewarding, as rice, maize, and wheat underpin global food security and account for a significant share of agricultural nitrogen demand.”

Box 2 The design–built–test–learn engineering cycle The design–build–test–learn cycle in plant synthetic biology serves as a framework for engineering increasingly complex biological traits. Given the sophisticated nature of metabolic networks, subcellular compartmentalization, and tissue-specific processes in plants, effective design requires a deep understanding of genetics and biochemical pathways. This knowledge informs rational decisions on pathway reconstruction, or even the development of entirely novel biosynthetic routes.

During the design phase, modular and standardized genetic parts are selected or engineered to optimize expression *in planta*. The build phase requires efficient DNA cloning systems that allow high-throughput, scarless and flexible assembly of multigene constructs. These cloning systems enable the efficient construction of transcriptional units. During the test phase, the assembled constructs are introduced into plant systems to assess their impact on the desired phenotype and overall plant fitness. The resulting data feeds into the learn phase, where performance metrics, unexpected interactions, and bottlenecks are analyzed to guide subsequent iterations.

Importantly, the cycle is iterative and capable of handling multiple constructs in parallel. The successful implementation of the design–built–test–learn cycle in plant engineering depends heavily on the speed of the plant transformation process and the developmental stage at which phenotypes can be quantified. The time required to generate transgenic plants can range from approximately two months in the case of Arabidopsis to over a year in more recalcitrant species. In the latter case, the number of possible iterations is significantly reduced. To address this limitation, much faster transient transformation systems are often used in parallel [Rizzo et al, (2023)].

## Perspectives on engineering nodulation

From a scientific perspective, it should be possible to replicate the evolutionary events that led to the development of nodulation. Doing so would provide a proof of principle for engineering nitrogen-fixing nodules in non-legume crops. By integrating fundamental knowledge from model legumes, nodulating non-legumes and the evolutionary trajectory of nodulation genes, researchers have formulated an initial blueprint for an engineering strategy [Huisman and Geurts (2020)], in which engineering high-affinity LCO receptors and rewiring the NIN transcriptional network are central components.

It has been demonstrated that non-nodulating plants can perceive rhizobium Nod factors, albeit with 1000-fold lower sensitivity than legumes. Crucially, it was shown that cereal LysM-type Nod factor receptors have the capacity to activate the nodule organogenesis program in legumes [Rübsam et al, (2023)]. With recent advances in characterizing the structural features of these LysM-type receptors, it is now a realistic goal to engineer their specificity and/or affinity toward rhizobium Nod factors in the target crop.

A critical challenge will be linking engineered Nod factor signaling to the transcriptional activation of a symbiotically functional *NIN* gene. NIN belongs to a small family of NIN-LIKE PROTEINS (NLPs), and most dicot plants, including Arabidopsis, possess putative *NIN* orthologs. Studies in this model plant have shown that NLPs act as nitrate sensors that are constitutively expressed in the root. Upon nitrate binding, NLPs translocate to the nucleus to activate nitrate-responsive downstream genes. This function is fundamentally different from that of symbiotic NIN, which is exclusively induced upon rhizobium Nod factor signaling and constitutively localized in the nucleus, activating symbiotic target genes [Liu and Bisseling (2020)]. All these differences represent ambitious engineering tasks, but none are insurmountable. If successful, this strategy could result in rhizobium-induced protonodules on non-legume crops.

Despite these groundbreaking discoveries that might enable successful engineering of nodule organogenesis, two aspects remain less well developed in the current engineering blueprint: enabling intracellular bacterial infection and creating a physiological framework that supports nitrogen fixation by rhizobia. Evolution has provided different possible paths for bacterial infection that could be engineered in non-legumes. Addressing these aspects requires further basic research to uncover critical mechanisms and evolutionary adaptations. This knowledge will help refine and enhance the engineering strategy. With current technological advancements, these bottlenecks can be overcome. Ultimately, this work may provide a positive answer to the once-naïve question posed by Burrill and Hansen in 1917: “*Is a symbiosis possible between legume bacteria and non-legume plants?*” [Burrill and Hansen (1917)].

“… two aspects remain less well developed in the current engineering blueprint: enabling intracellular bacterial infection and creating a physiological framework that supports nitrogen fixation by rhizobia.”

## Supplementary information

Peer Review File
