# Supplementary material for: Self-fertilizing crops: Plant Biotech’s Hardest, Most Rewarding Task
Source: EMBO Rep. 2025 Nov 11;26(23):5644–8. doi: 10.1038/s44319-025-00618-y (PMC12678418; doi:10.1038/s44319-025-00618-y)
Supplement: Supplementary file 1 — Peer Review File [file 44319_2025_618_MOESM1_ESM.pdf]

EMBO reports

Self-Fertilizing Crops. Plant Biotech's Hardest, Most Rewarding Task

Rene Geurts and Simona Radutoiu

Corresponding author(s): Rene Geurts (rene.geurts@wur.nl)

Review Timeline:

Submission Date:	14th Oct 25
Accepted:	20th Oct 25

Editor: Holger Breithaupt

Transaction Report: This article was editorially reviewed.
